# Metformin Treatment Modulates Long Non-Coding RNA Isoforms Expression in Human Cells

**DOI:** 10.3390/ncrna8050068

**Published:** 2022-10-12

**Authors:** Izabela Mamede C. A. da Conceição, Thomaz Luscher-Dias, Lúcio R. Queiroz, Ana Gabrielle B. de Melo, Carlos Renato Machado, Karina B. Gomes, Renan P. Souza, Marcelo R. Luizon, Glória R. Franco

**Affiliations:** 1 Departamento de Bioquímica e Imunologia, Instituto de Ciências Biológicas, Universidade Federal de Minas Gerais, Belo Horizonte 31270-901, MG, Brazil; 2 Departamento de Análises Clínicas e Toxicológicas, Escola de Ciências Farmacêuticas, Universidade de São Paulo, São Paulo 05508-000, SP, Brazil; 3 Departamento de Análises Clínicas e Toxicológicas, Faculdade de Farmácia, Universidade Federal de Minas Gerais, Belo Horizonte 31270-901, MG, Brazil; 4 Departamento de Genética, Ecologia e Evolução, Instituto de Ciências Biológicas, Universidade Federal de Minas Gerais, Belo Horizonte 31270-901, MG, Brazil

**Keywords:** lncRNA, metformin, transcriptome, RNA isoforms

## Abstract

Long noncoding RNAs (lncRNAs) undergo splicing and have multiple transcribed isoforms. Nevertheless, for lncRNAs, as well as for mRNA, measurements of expression are routinely performed only at the gene level. Metformin is the first-line oral therapy for type 2 diabetes mellitus and other metabolic diseases. However, its mechanism of action remains not thoroughly explained. Transcriptomic analyses using metformin in different cell types reveal that only protein-coding genes are considered. We aimed to characterize lncRNA isoforms that were differentially affected by metformin treatment on multiple human cell types (three cancer, two non-cancer) and to provide insights into the lncRNA regulation by this drug. We selected six series to perform a differential expression (DE) isoform analysis. We also inferred the biological roles for lncRNA DE isoforms using in silico tools. We found the same isoform of an lncRNA (AC016831.6-205) highly expressed in all six metformin series, which has a second exon putatively coding for a peptide with relevance to the drug action. Moreover, the other two lncRNA isoforms (ZBED5-AS1-207 and AC125807.2-201) may also behave as cis-regulatory elements to the expression of transcripts in their vicinity. Our results strongly reinforce the importance of considering DE isoforms of lncRNA for understanding metformin mechanisms at the molecular level.

## 1. Introduction

Metformin is the first-line glucose-lowering drug most commonly used to manage hyperglycemia in patients with type 2 diabetes mellitus [1]. It has also been used as a treatment for several other metabolic diseases including polycystic ovary syndrome [2,3] and gestational diabetes [4], and for obesity in children and adolescents [5].

The molecular mechanisms underlying metformin action are not yet fully understood. Metformin has a potent anti-proliferation action, which is commonly observed but not thoroughly explained [6,7,8]. The liver is the major target for metformin, where it inhibits gluconeogenesis by activating the AMP-activated protein kinase (AMPK) pathway [9,10]. Metformin has also been suggested to inhibit hepatic gluconeogenesis in an AMPK pathway-independent way [11]. The AMPK-dependent and AMPK-independent mechanisms underlying metformin action are reviewed elsewhere [12]. Metformin is also known to act in several other tissues, affecting tumorigenesis by reducing energetic stress and altering tumor progression by systemic decrease of insulin and immunometabolic pathway activities [13,14,15]. Nonetheless, there are several phase II and phase III clinical studies aiming to repurpose metformin for use in cancer treatment (https://clinicaltrials.gov/ct2/results?cond=metformin&Search=Apply&age_v=&gndr=&type=&rslt=&phase=1&phase=2, accessed on 5 August 2022)

A comprehensive genomic characterization of metformin response on human primary hepatocytes was performed using RNA-seq and ChIP-seq to identify genes and regulatory elements associated with metformin action [16]. Other global transcriptomic analyses using metformin in different cell types have also been conducted [17,18,19]. However, these previous studies focused only on protein-coding genes.

Long noncoding RNAs (lncRNAs) play a key role in the fine regulation of gene expression [20,21] by acting through their transcribed form and, occasionally, by coding for micropeptides using non-canonical translation pathways [22]. They are, for the most part, transcribed by RNA polymerase II and therefore have a poly-adenylated tail, which distinguishes them from the other types of well-studied regulatory RNAs, as microRNAs [23]. lncRNAs can act on the same locus where they are transcribed (cis-acting), in distant loci (trans-acting), or outside the nucleus [24,25]. The expression of individual lncRNAs in response to drug treatments has been explored recently, but global studies of differential expression of lncRNAs in response to drugs are still scarce [26].

More than 90% of all human genes undergo alternative splicing which, along with alternative transcription start and end points, is one of the primary producers of multiple transcript isoforms [27]. When compared to protein-coding genes, lncRNAs possess a higher number of isoforms transcribed from the same genomic region [28]. Notably, metformin directly affects genes for proteins of the spliceosome and regulates the splicing of several other genes [17,29]. Nevertheless, differential transcript expression is rarely analyzed, especially for noncoding genes, and only a few well-studied lncRNAs are routinely analyzed at the isoform level, such as *NEAT1* [30].

To date, no previous study has performed a global identification of lncRNAs affected by metformin treatment nor explored its ability to regulate gene expression at the transcript level. In this study, we aimed to globally characterize the lncRNA isoforms that were differentially regulated by metformin on human cell types (four cancer cells, one primary cell, and two embryonic stem cells) using openly public transcriptomic data, and to provide insights into the possible roles of these molecules.

## 2. Results

### 2.1. Metformin Treatment Promotes Differential Expression of lncRNA Isoforms among Cell Lines

**Figure 1:**
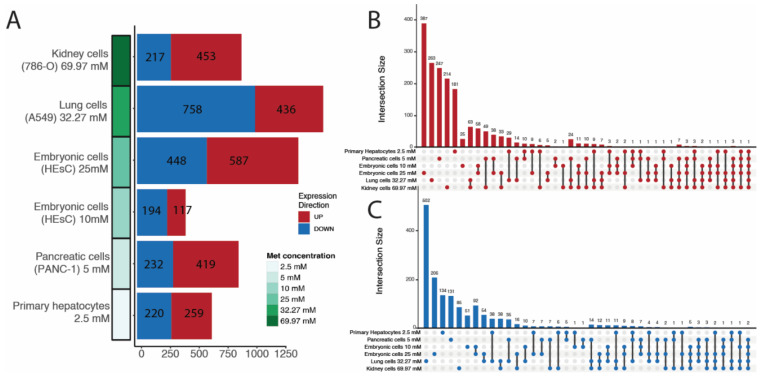
(**A**) Differential lncRNA isoforms counted per series using a cutoff of *p*-value < 0.05 and absolute log2foldchange > 0.5. Series are ordered according to descending metformin concentration. Number of upregulated lncRNA isoforms shown in dark red and number of downregulated lncRNA isoforms shown in dark blue. Upset plots of up- (**B**) and downregulated (**C**) lncRNA isoforms which intersect between series.

**Figure 2:**
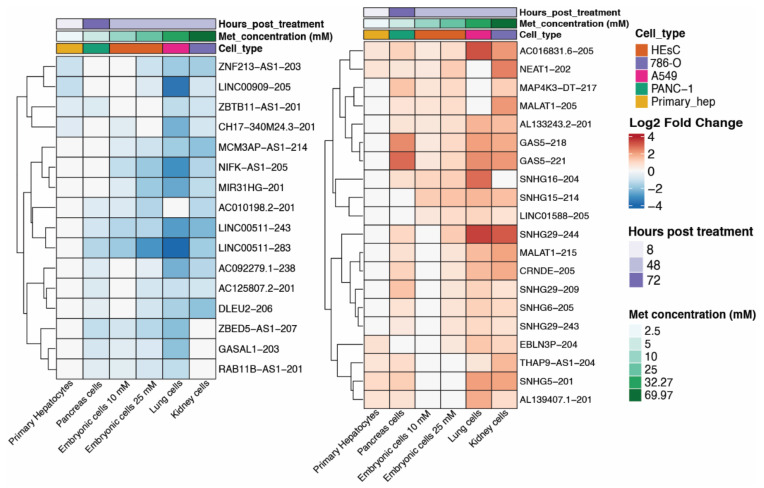
Heatmap of the 36 differentially expressed isoforms which are present in at least four of the six series. Heatmap was ordered according to Metformin concentration (lowest to highest, in green) and information on hours posttreatment (in purple) and cell type are present in the top. Color intensity represents log2FoldChange in the transcript in each series. Upregulated transcripts are in red, and downregulated transcripts are in blue. Panc-1: human pancreatic cell line isolated from pancreatic carcinoma. HeSC: human embryonic stem cells. 786-O: renal cell carcinoma. A549: human lung epithelial carcinoma cells. Primary hep: primary human hepatocytes.

The results show that 2271 isoforms were upregulated and 2069 were downregulated in cells treated with metformin. Embryonic cells treated with 25 mM of metformin had the highest number of upregulated transcripts (*n* = 587), while lung cells treated with 32 mM of metformin had the highest number of downregulated transcripts (*n* = 758) (Figure 1A). Although the number of differentially expressed lncRNA isoforms varied among cell lines, covariance analysis did not show any significant correlation of isoform count to sequencing depth, metformin dose, or hours posttreatment (Figure S2). Most DE lncRNA isoforms were cell line-specific, and the pairs of cell lines with the most common isoforms were those from the same experiment, such as embryonic cells treated with 10 and 25 mM metformin and lung and kidney cells (Figure 1B,C). A few lncRNA isoforms (74 upregulated and 84 downregulated) were present in at least three series. An even lower number are present in at least four, where 20 are upregulated and 16 downregulated. Only four lncRNA are commonly upregulated in five series, while two are downregulated, and only one upregulated isoform is present in all six series. Since our focus was to explore the common effect of metformin on lncRNA isoform expression, subsequent analyses were based on the 36 isoforms that were altered in response to the drug treatment in at least four of the six series (Figure 2). It is worth noting that the series with highest metformin concentration also possess the highest number of shared DE isoforms. The same was not seen in the series with the lowest metformin concentration. Variations in DE lncRNA isoforms among series can be related to cell type, library depth, or hours posttreatment difference.

### 2.2. Different Isoforms of lncRNAs Are Modulated by Metformin Treatment

**Figure 3:**
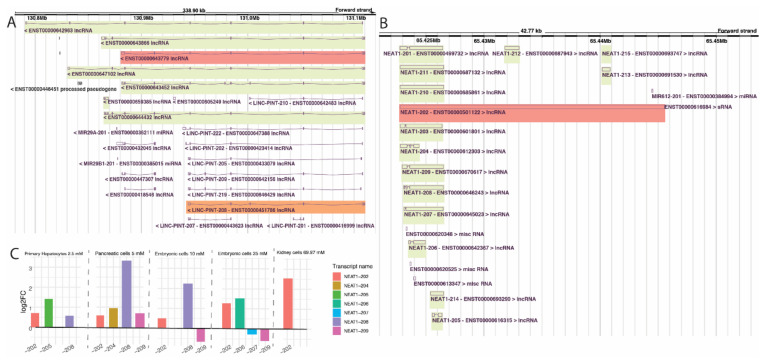
(**A**) region around the AC016831.6-205 transcripts and its superposition with LINC-PINT-208. In A, region of some of the many annotated LINC-PINT and AC016831.6-205 transcripts; in B, approximation and superposition of the mentioned isoforms. In red, the AC016831.6 isoform encountered in our analysis; in green, other isoforms of the same gene; in orange, the LINC-PINT isoform associated with the one we found. (**B**) NEAT1 genomic region with its transcripts from *Ensembl Genome Browser*. In red, NEAT1-202, the isoform encountered in our data, which is also the canonic NEAT1 isoform. In green, all other NEAT1 isoforms. (**C**) NEAT1 isoforms differential expression per series. Bar length represents log2FoldChange in each isoform. Series are ordered according to metformin concentration, and NEAT isoforms are colored according to legend at the right side.

AC016831.6-205 (ENST00000604514.1) was the only isoform upregulated in all six cell lines, and no isoform was downregulated in all series. AC016831.6-205 is a recently annotated transcript on chromosome 7 that shares the same genomic locus with transcripts of the Long Intergenic Non-Protein Coding RNA P53 Induced, *LINC-PINT* (ENSG00000231721.7), and might be a novel isoform of that gene (Figure 3A). The closest *LINC-PINT* isoform to AC016831.6-205 is the isoform 208. A back-splicing event circularizes the second exon of LINC-PINT-208, and, in the cytoplasm, this circular RNA codes for a peptide originated from an internal ribosome entry site (IRES). Upregulation of this peptide is a marker of beneficial prognosis in specific cancer types [31]. It is worth noting that metformin has been suggested as a potential therapeutic approach for multiple types of cancer [13,32].

Of the 36 lncRNA isoforms affected in at least four cell lines, four are upregulated, and two are downregulated in the five series. The four upregulated isoforms are NEAT1-202 (ENST00000501122.2), GAS5-218 (ENST00000448718.5), GAS5-221 (ENST00000450589.5), and the novel annotated transcript AL133243.2-201 (ENST00000610331.1). *NEAT1* has been previously found perturbed (up or downregulated) at the gene-level in metformin-treated cells [33], and the predicted function of this gene in response to treatment can differ depending on the cancer cell line [34]. Notably, our study is the first to analyze *NEAT1* at isoform level and in association with metformin treatment. The NEAT1-202, upregulated in five out of six cell lines in our study, is the canonical isoform of the gene (Figure 3B), having a more extended downstream region compared to the noncanonical isoforms of *NEAT1* (Figure 3B). *NEAT1* currently has 15 annotated isoforms, of which six were found differentially expressed in at least one cell line, with different fold change directions (Figure 3C). Our results suggest that the conflicts in metformin-induced *NEAT1* expression from previous studies could be due to the expression analyses being performed only at the gene level, thus disregarding the expression of different isoforms of the lncRNA at the transcript level.

Changes in the *GAS5* gene expression have not been previously associated with metformin treatment. Nevertheless, there are multiple studies associating the expression of this lncRNA with diseases that are currently treated with metformin, including diabetes [35], cardiovascular diseases [36], and cancer [37]. *GAS5* has over 70 annotated isoforms and is one of the lncRNAs with the highest number of transcripts. GAS5-218 and GAS5-221, upregulated in five out of six cell lines, share most of their exons and introns, only differing in the length of the seventh exon (Figure S3).

The only lncRNA isoforms that appeared downregulated in at least five cell lines are isoforms of the Long Intergenic Non-Protein Coding RNA 511: LINC00511-243 (ENST00000648623.2) and LINC00511-283 (ENST00000650131.2) (Figure S4). Changes in the *LINC00511* gene expression have also not been previously associated with metformin treatment; however, similar to *GAS5*, most conditions that *LINC00511* was previously associated with can be related to metformin [38,39]. *LINC00511* is a poor prognostic factor in cancer since its increased expression is crucial to malignant tumorigenesis [40]. *LINC00511* presents multiple oncogenic functions, including the promotion of cellular proliferation, acceleration of metastasis, and influence on the invasive behavior of cancer cells [41]. Although *LINC00511* has over 100 different annotated transcripts, this gene had never been analyzed at the transcript level in any high-throughput transcriptomic study. The downregulated transcripts found in this study have a smaller downstream of gene (DoG) region when compared to the canonical LINC00511-244 isoform (Figure S3B). LINC00511-243 also has a different second exon compared to the canonical isoform (Figure S3B).

All 36 DE lncRNA transcripts (Table S2) present in at least four cell lines were then selected for further analyses to detect possible cis- and trans-acting mechanisms of action.

### 2.3. Metformin Regulates Cis-Acting lncRNA Isoforms

**Figure 4:**
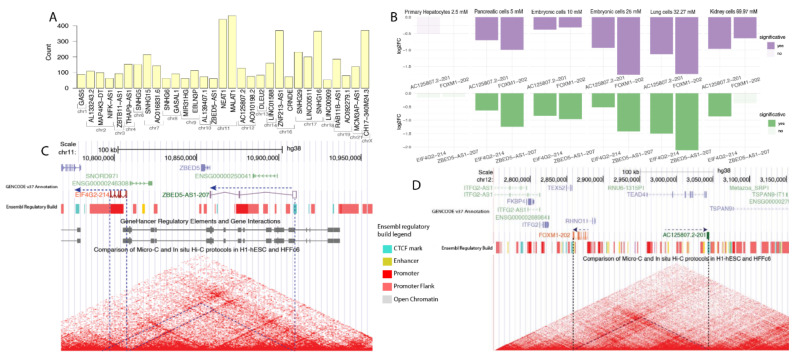
(**A**) total transcript counts in the 1 mb to each side region around the lncRNA for the 36 lncRNA selected for further analysis. (**B**) expression bar plots of the lncRNA isoform—transcript pairs which passed on the correlation cutoff (corr > 0.8 and *p*-value < 0.01) and on the genomic distance cutoff (1 mb to each side). On the top, AC125807.2-201 (ENST00000513358.3) and FOXM1-202 (ENST00000359843.8); on the bottom, ZBED5-AS1-207 (ENST00000664276.1) and EIF4G2-204 (ENST00000530211.6). Significance is computed as values inferior to 0.05. (**C**) Genomic region of around 300kilobases in chromosome 12. Tracks: GENCODE v37 annotation, ENCODE Hi-C, and *Ensembl* regulatory elements data. (**D**) genomic region of around 150kilobases in chromosome 11. Tracks: GENCODE v37 annotation, ENCODE Hi-C, *Genehancer* IM-PET, and *Ensembl* regulatory elements data.

LncRNAs can exert their roles through cis- or trans-acting mechanisms [42]. A cis-acting effect occurs when an lncRNA interacts with DNA or proteins in the locus vicinity from where the gene was transcribed. These interactions usually affect the expression of other genes embedded within this region or in regions within the exact topologically associated domains (TADs) [43]. To analyze the potential cis-acting effects of the selected lncRNAs on the expression of other genes, we performed a correlation analysis between the expression of lncRNAs and that of transcripts encoded by genes located up to 1 mega-base (Mb) down- or upstream of the selected lncRNAs (Figure 4A). This range was selected because it corresponds to the highest median length of human TADs, as previously described by Winick-Ng et al. [44].

*MALAT1* and *NEAT1* were the lncRNAs with the highest number of neighbor DE transcripts following metformin treatment: 458 and 437, respectively (Figure 4A). Both these lncRNAs are located in a very populated region on chromosome 11. Conversely, *LINC00909* and *ZBED5-AS1* were the lncRNAs with the lowest number of DE transcripts in their vicinity, with fewer than 100 transcripts around their genomic position (Figure 4A).

Two transcripts of genes neighboring metformin-affected lncRNAs presented a positive expression correlation, with transcripts of those lncRNAs (Pearson R > 0.8 and *p*-value < 0.01): AC125807.2-201 (ENST00000513358.3) correlated with FOXM1-202 (Forkhead box M1 isoform 202) (ENST00000359843.8) and ZBED5-AS1-207 (ENST00000664276.1) correlated with EIF4G2-204 (Eukaryotic translation initiation factor 4 gamma 2) (ENST00000530211.6) (Table S3). AC125807.2 is a recently annotated lncRNA on chromosome 12 which possess only one isoform, AC125807.2-201, that is downregulated in five series (Figure 4B, top panel). This lncRNA isoform is co-expressed with FOXM1-202, the canonical *FOXM1* isoform commonly overexpressed during mitosis and in cancer progression [43,45]. AC125807.2-201 overlaps with a CTCF (CCCT binding factor) mark and is positioned on the border of a TAD, which also includes the *FOXM1* gene (Figure 4C). This finding suggests that AC125807.2-201 could be acting as a topological anchor point RNA (tapRNA), a recently proposed class of lncRNAs which are located at chromatin anchor points and on the border of TADs, regulating their activity [43]. Interestingly, *FOXM1* previously was found to be downregulated in metformin-treated cells [46,47,48]. Our results suggest that AC125807.2-201 may act as a regulator for FOXM1-202 expression in a cis-acting manner.

The other pair of lncRNA isoform and co-expressed transcript which passed on the significance level in the correlation analyses consisted of lncRNA ZBED5-AS1-207 (ENST00000664276.1) and EIF4G2-214 (Eukaryotic Translation Initiation Factor 4 Gamma 2 isoform 214) (ENST00000530211.6), which are both located on chromosome 11. *ZBED-AS1* is an antisense lncRNA of *ZBED5* (Zinc Finger BED-Type Containing 5), and it appears downregulated in four of the six series analyzed. ZBED-AS1-207 and EIF4G2-214 are located less than 50 kb apart from each other and belong to the same TAD (Figure 4D). There is a predicted regulatory element interaction between the last exon of ZBED-AS1-2017 and the beginning of EIF4G2-214 (Figure 4D). *EIF4G2*, previously known as *DAP5*, is a homolog of *EIF4G1* and is not part of the canonical translation process, even though deletion of this gene disrupts embryogenesis [49,50]. *EIF4G2* is related to the alternative scanning of the translation start site by the 40S ribosomal subunit, translation associated with alternative ORFs, and translation under stress [51,52]. *EIF4G2* differential expression was previously related to extracellular matrix remodeling [53]. However, most of the literature regarding this gene explores its interaction with microRNAs, and no metformin effect on *EIF4G2* expression has been previously reported. Notably, EIF4G2-214 is not the canonical *EIF4G2* isoform. EIF4G-214 loses most of its predicted active protein-coding domains, with only part of the MIF4G superfamily coding portion remaining (Figure S5). This domain is responsible for RNA and for DNA binding of the EIF4 family [54], and its loss suggests that EIF4G-214-translated peptide is working as a competitor for the canonical protein to bind to DNA and RNA. Thus, our results suggest that ZBED-AS1-207 may control the expression of EIF4G2-214 in a cis-acting manner.

### 2.4. Metformin Regulates Trans-Acting lncRNA Isoforms

LncRNAs can also regulate gene expression across large genomic distances and on other chromosomes [55]. They can act in the form of RNAs by interacting with proteins, RNAs, or other components of the cell machinery to regulate their activity and function [41]. Alternatively, lncRNAs can also be translated into micro-peptides that, in turn, interact with other proteins or RNAs [22]. Trans-acting effects are defined as these roles played by lncRNAs away from the region where they are transcribed [23].

**Figure 5:**
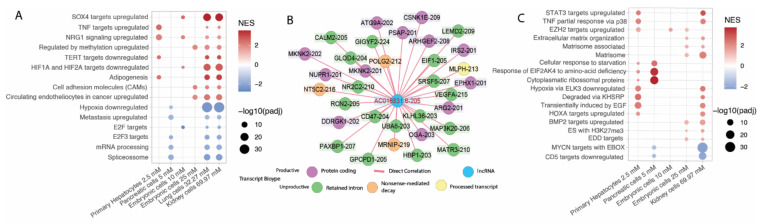
(**A**) enrichment of AC016831.6-205 (ENST00000604514.1) targets using *fgsea* algorithm and the MsigDB C2 pathway dataset. NES was calculated using correlation values as the ranking; thus, positive NES indicates direct correlation between those target-transcripts and the pathway, and negative NES indicates inverse correlation between those target-transcripts and the pathway. Only protein-coding transcripts were used as target genes for this analysis. The use cutoff was *p*-adjusted inferior to 0.0001. x axis ordered according to series metformin concentration. (**B**) Network representation of target genes of AC016831.6-205 (ENST00000604514.1) are differentially expressed transcripts in at least 4 series and pass on the correlation cutoff (corr > 0.8 and *p*-value < 0.01). (**C**) enrichment of NEAT1-202 targets using *fgsea* algorithm and MsigDB C2 pathway dataset. Other information same as 5A.

Notably, AC016831.6-205 (ENST00000604514.1) is the only lncRNA isoform that is upregulated in all metformin-treated cells. The second exon of this transcript can encode an anti-oncogenic peptide [31], as discussed above. This putative peptide, which might arise due to the circularization and translation of exon 2, has been characterized elsewhere [31] but not further explored. Functional enrichment analysis of all possible target genes of AC016831.6-205 was performed, ranking those potential targets by correlation value. We calculated a normalized enrichment score for known biological pathways enriched for the genes with transcripts positively or negatively correlated with AC016831.6-205 (Figure 5A). The resulting pathways corroborate the possible trans-action function of this RNA and the known effects of metformin (Figure 5A), such as the enrichment of HIF1A and HIF2A (hypoxia-induced factor 1A and 2A) pathways and hypoxia pathways. Hypoxia regulation is related to metformin-increased activity of the mitochondrial complex I [56,57]. Pathways associated with proliferation, such as adipogenesis (positive correlation) and metastasis (negative correlation), were also enriched among transcripts correlated with AC016831.6-205 (Figure 5A).

Recently, researchers have described a potential effect of metformin on splicing [17] similar to that seen for other drugs [58,59]. Here, we show that the mRNA processing and spliceosome pathways negatively correlate with AC016831.6-205 expression (Figure 5A), suggesting that an increase of the AC016831.6-205 expression may regulate the metformin effect of reducing splicing. Supporting these results, several DE transcripts that showed a direct correlation with AC016831.6-205 have retained introns or contain premature stop-codons that render them suitable for nonsense-mediated decay (Figure 5B). Three of those retained intron transcripts (CALM2-205, VEGFA-215, and MAP3K20-206) are essential in metformin biological roles, such as *CALM2* that acts on proliferation and cell signaling [60,61], *VEGFA* that acts as a vascular growth factor [62,63], and *MAP3K20* that acts in cell-cycle arrest checkpoints [64]. For instance, metformin was previously found to upregulate *VEGFA* nonprotein coding isoforms [29]. *VEGFA* is a gene associated with the proliferation and migration of endothelial cells [62]. The effect of metformin on this gene might be relevant to the current uses of the drug as an anti-proliferation agent. Metformin does not disrupt *VEGFA* by downregulating the expression of the gene but rather by influencing its alternative splicing and the production of nonprotein coding isoforms of the gene in a model for diabetic retinopathy [29]. We suggest that this dysregulation may be associated with AC016831.6-205 expression per se or its effect on splicing.

NEAT1-202 is overexpressed in five of the six series. Extracellular matrix remodeling pathways were among those pathways that were enriched for transcripts positively correlated with NEAT1-202 (Figure 5C). *NEAT1* has been previously associated with matrix remodeling, but with contradictory results depending on the analysis method used. Several studies associated NEAT1 overexpression with fibrosis progression and also with fibrosis reduction [65,66,67,68]. Nevertheless, studies of matrix remodeling did not analyze *NEAT1* at the isoform level.

## 3. Discussion

As far as we know, this is the first study that analyzed lncRNA expression in response to metformin across distinct cell lines and drug doses at the transcript level. Our integrative RNA-seq analysis of multiple cell lines treated with metformin shows that this drug regulates critical isoforms of lncRNAs, which are closely connected to the proposed actions of the drug. For instance, we point to the lncRNA isoform AC016831.6-205, which was overexpressed in all cell lines, as a potential noncoding marker of metformin treatment. The second exon in AC016831.6-205 may code for a micro-peptide with potential biological function related to the effects of metformin in cancer. We also show that metformin might induce the expression of lncRNA isoforms, such as ZBED5-AS1-207 and AC125807.2-201, with putative cis-acting effects on drug-related genes, including FOXM1-202 and EIF4G2-214. These genes might play a role in the metformin effect on proliferation and energy metabolism [8,10,32].

Previous studies provide unclear findings regarding metformin actions. These studies were conducted with supra-pharmacological concentrations (doses) of metformin that were 10–100 times higher than the therapeutic dose recommended to patients with type 2 diabetes mellitus [69]. Indeed, at the molecular level, the findings vary depending on the metformin doses and treatment duration, with apparent differences between acute and chronic administration [8]. Our study was also conducted with supra-pharmacological doses of metformin, but the literature suggests that primary exploration gene expression studies should be conducted with these doses [70].

Most of the lncRNAs we highlighted here have not previously been associated with metformin. LncRNAs have a lower expression level compared to protein-coding genes [21]. This amount is even lower when we analyze them at the isoform-specific level [71]. Since most studies accomplish gene-level expression analysis and use high fold change cutoffs and analysis methods which are unsuited for lncRNA isoform detection, most lncRNA expression might have been missed in previous works.

Characterizing differentially expressed lncRNAs in multiple cell types is a disadvantage in this work, since we are potentially ignoring lncRNAs with cell-type specific expression, which is a common feature of these genes [72]. Nevertheless, as an exploratory analysis of commonality in drug action in different cells, we could still find the same transcript, AC016831.6-205, upregulated in all cell types, despite the considerable heterogeneity among them, showing the potential for a cell type-independent marker. However, since isoform level quantification is rare in previously published works, we could not find other studies that used all annotated lncRNA isoforms for differential expression analysis. Despite the cell-type heterogeneity, metformin concentration, and hours posttreatment among the samples, our standardized criteria and our usage of a lncRNA detection tailored workflow allowed for a reliable comparison between sequencing libraries. For this reason, we focused our analysis on common isoforms expressed in all cells.

Our analysis of the cis-acting lncRNAs, as seen on the co-expressed pair ZBED5-AS1-207 and EIF4G2-214 and the likely peptide encoded by the circularization, followed by IRES-mediated translation of exon 2 of AC016831.6-205, point to a potential role of metformin in regulating alternative translation in treated cells. Cancer cells are known to switch cell translation to alternative modes [73], but other types of environmental stress, such as drug treatment, have not been previously associated with this switch on cell translation. We suggest that the role of metformin in reducing cancer proliferation could be related to the potential of this drug to induce alternative translation. The hyperexpression of AC016831.6-205 in all cell lines is interesting, since this lncRNA isoform has not been previously associated with any phenotype in the literature.

Mice lncRNAs have been previously associated with metformin treatment accessed by RT-qPCR and microarray analyses [74,75,76]. Nevertheless, these types of analyses do not allow isoform-specific evaluation due to technical limitations and also because of the less extensive ncRNA annotation of mouse if compared to human genome. Other types of regulatory ncRNA, like microRNA, have been formerly demonstrated as affected by metformin treatment. As an example, the miR-195, which has been identified in patients’ peripheral blood, has been shown to be capable of regulating a vast number of genes, mediating the knockdown of their mRNA. [77] Multiple microRNAs which are important in diabetic related conditions have also been identified in the blood of patients [77,78,79]. It is noteworthy to point out that the RNA-seq studies analyzed here utilized RNA that was captured by the polyadenylated tail. So, we were not able to find microRNAs in these transcriptomes, nor are any of our lncRNA candidates already predicted as sponges to microRNAs in the literature.

In silico only functional characterization of lncRNA function is widely discussed in the literature, such as with other ncRNAs [80]. As to limitations of the present study, we are aware that our results are not sufficient to prove the cis- and trans-acting regulation by the lncRNAs on the suggested genes, which would only be possible with experimental approaches. However, we were able to broaden the knowledge of several lncRNAs potentially associated with metformin treatment and its effects in human diseases, including cancer. Moreover, our results build upon and broadly advance on the goal set by the studies’ authors, which produced the RNA-seq data analyzed here. The lung and pancreas study aimed to observe metformin and aspirin combined action in cancer cells [18]. The pancreas study aimed to examine the metformin effect on GPD-deficient cells [19]. The embryonic cell study aimed to characterize the metformin effect on spliceosome-related transcripts [17]. The only study which aimed to characterize the molecular actions of metformin was the primary hepatocyte study, but it did not identify DE lncRNAs [16].

In conclusion, we identified lncRNA isoforms which may be crucial to the metformin cellular mechanism of action and the biological processes in which they may be acting. We also suggested possible functions for these lncRNA isoforms and distinguished molecular effects between lncRNA isoforms transcribed from the same gene. Additionally, we showed that the heterogeneity arising from different cell types and drug concentrations produces different molecular signatures in response to metformin.

## 4. Materials and Methods

### 4.1. Data Selection

We searched for all experiments with public raw data with unrestricted use available on the Sequence Read Archive (SRA) using a systematic search method and “metformin” [MesH] as the main keyword. All human RNA-seq libraries obtained in this search were selected for further filtering. From these libraries, we included those with a read length of 100 or 150 bases and a sequencing depth of at least 20 million reads, which were sequenced using Illumina technologies with paired-end libraries and presented paired-case and control triplicates, with the polyA capture method. Six libraries from four different studies were included (SRA projects: PRJNA277028, PRJNA324847, PRJNA577137 and PRJNA612620) (Figure S1 and Table S1). A correlation analysis was performed for continuous series characteristics using the Pearson test (Figure S2).

### 4.2. Transcript Expression Quantification

Transcript-level quantification was performed from the raw RNA-seq reads using the pseudo-alignment software Salmon [81] and the GENCODE v37 [82] human reference transcriptome. Salmon was run multiple times for results comparison and settings test. The index was created with k-mer of size 31 nt, and quantification was run on default settings plus the following flags: —*validateMappings*, —*numGibbsSamples 100*, —*gcBias*, and —*seqBias*. GENCODE v37 was also used to construct a transcript to a gene dictionary containing the biotypes of transcripts and genes. The *tximeta* R package [83] was used to import Salmon results to R and summarize them in transcript and gene counts (Figure S1).

### 4.3. Differential Transcript Expression Analysis

Differential expression was individually calculated for each series from transcript and gene counts using the *swish* function of the *fishpond* R package [84]. Metformin and paired control groups were the contrast group used in each comparison, resulting in six independent expression tables (Table S2). The cutoff for differential expression was *p*-value inferior to 0.05 and log2FoldChange superior to 0.5. We used this less stringent cutoff compared to that commonly used for protein-coding genes and transcripts, since lncRNAs have lower expression levels and, therefore, lower absolute fold changes. Differentially expressed lncRNA isoforms were then compared among experiments, and all isoforms present in at least four out of six experiments were selected for further analysis (Figure S1).

### 4.4. Transcript Expression Correlations

Expression correlation was performed using transcripts per million counts outputted from the *tximeta* and *Rcorr* packages using a *t*-test after Pearson coefficient correlation estimation. The correlation was estimated from the differentially expressed transcripts (DETs) in four or more series and all other DETs, and between those DETs and all transcripts present in the transcripts per million (TPM) counting tables. Correlation cutoff was established in 0.8 (80%) correlation value and 0.01 *p*-value (Figure S1, Table S3).

### 4.5. Functional Annotation Analyses and Genomic Region Filters

Functional annotation and genomic filtering were performed using *GenomicRanges* and *biomart* R packages. *Ensembl* and ENCODE tracks were used as input data to both *Ensembl Genome Browser* and *UCSC Genome Browser*. Cis-acting lncRNA distances were measured using topologically associating domain (TAD) length estimates from other published data [44]. The final cutoff was 1000 kilobases flanking the gene region (Figure S1).

### 4.6. Functional Enrichment Analyses

Functional enrichment analyses were conducted using the *fgsea* R package for possible trans-acting lncRNA targets. *fgsea* works by comparing all present transcripts and using a ranking value to find enriched gene sets. The selected ranking stat was the medium correlation value of the chosen lncRNA isoform and each other transcript in the analysis per series. The used gene sets were obtained from the molecular signature database (MSigDB) C2 curated gene sets [85]. Enrichment cutoff was considered as FDR-adjusted *p*-values ranging from 0.0001 to 0.001, presence in at least two independent experiments, and suggested biological association with Metformin actions. Enrichment tables with only the FDR adjusted *p*-values cutoff can be found in Table S4.

## Data Availability

This study did not provide any new submitted data.

## Supplementary Materials

The following supporting information can be downloaded at: https://www.mdpi.com/article/10.3390/ncrna8050068/s1, Figure S1: Global vision of the used pipeline; Figure S2: Pearson correlation map between number of upregulated and downregulated isoforms and other experiment characteristics; Figure S3: GAS5 genomic region with its transcripts from Ensembl genome Browser; Figure S4: LINC00511 genomic region with some of its transcripts from Ensembl Genome Browser; Figure S5: Predicted domains of EIF4G2-201 and EIF4G2-214; Table S1: Characteristics of the libraries used; Table S2: Differential Expression results for each series; Table S3: Correlation for all DETs; Table S4: Functional Enrichment for NEAT1-202 and AC016831.6-205 in each series.

## References

[1] Zaccardi F., Khunti K., Marx N., Davies M.J. (2020). First-line treatment for type 2 diabetes: Is it too early to abandon metformin?. Lancet.

[2] Cao Q., Hu Y., Fu J., Huang X., Wu L., Zhang J., Huang W. (2021). Gestational metformin administration in women with polycystic ovary syndrome: A systematic review and meta-analysis of randomized control studies. J. Obstet. Gynaecol. Res..

[3] Magzoub R., Kheirelseid E.A.H., Perks C., Lewis S. (2022). Does metformin improve reproduction outcomes for non-obese, infertile women with polycystic ovary syndrome? Meta-analysis and systematic review. Eur. J. Obstet. Gynecol. Reprod. Biol..

[4] Li C., Gao C., Zhang X., Zhang L., Shi H., Jia X. (2022). Comparison of the effectiveness and safety of insulin and oral hypoglycemic drugs in the treatment of gestational diabetes mellitus: A meta-analysis of 26 randomized controlled trials. Gynecol. Endocrinol..

[5] Masarwa R., Brunetti V.C., Aloe S., Henderson M., Platt R.W., Filion K.B. (2021). Efficacy and Safety of Metformin for Obesity: A Systematic Review. Pediatrics.

[6] Foretz M., Guigas B., Viollet B. (2019). Understanding the glucoregulatory mechanisms of metformin in type 2 diabetes mellitus. Nat. Rev. Endocrinol..

[7] LaMoia T.E., Butrico G.M., Kalpage H.A., Goedeke L., Hubbard B.T., Vatner D.F., Gaspar R.C., Zhang X.M., Cline G.W., Nakahara K. (2022). Metformin, phenformin, and galegine inhibit complex IV activity and reduce glycerol-derived gluconeogenesis. Proc. Natl. Acad. Sci. USA.

[8] Rena G., Hardie D.G., Pearson E.R. (2017). The mechanisms of action of metformin. Diabetologia.

[9] Hundal R.S., Krssak M., Dufour S., Laurent D., Lebon V., Chandramouli V., Inzucchi S.E., Schumann W.C., Petersen K.F., Landau B.R. (2000). Mechanism by which metformin reduces glucose production in type 2 diabetes. Diabetes.

[10] Zhou G., Myers R., Li Y., Chen Y., Shen X., Fenyk-Melody J., Wu M., Ventre J., Doebber T., Fujii N. (2001). Role of AMP-activated protein kinase in mechanism of metformin action. J. Clin. Investig..

[11] Foretz M., Hebrard S., Leclerc J., Zarrinpashneh E., Soty M., Mithieux G., Sakamoto K., Andreelli F., Viollet B. (2010). Metformin inhibits hepatic gluconeogenesis in mice independently of the LKB1/AMPK pathway via a decrease in hepatic energy state. J. Clin. Investig..

[12] Foretz M., Guigas B., Bertrand L., Pollak M., Viollet B. (2014). Metformin: From mechanisms of action to therapies. Cell Metab..

[13] Ahmed Z.S.O., Golovoy M., Abdullah Y., Ahmed R.S.I., Dou Q.P. (2021). Repurposing of Metformin for Cancer Therapy: Updated Patent and Literature Review. Recent Pat. Anticancer Drug Discov..

[14] Ma R., Yi B., Riker A.I., Xi Y. (2020). Metformin and cancer immunity. Acta Pharmacol. Sin..

[15] Pernicova I., Korbonits M. (2014). Metformin—Mode of action and clinical implications for diabetes and cancer. Nat. Rev. Endocrinol..

[16] Luizon M.R., Eckalbar W.L., Wang Y., Jones S.L., Smith R.P., Laurance M., Lin L., Gallins P.J., Etheridge A.S., Wright F. (2016). Genomic Characterization of Metformin Hepatic Response. PLoS Genet..

[17] Laustriat D., Gide J., Barrault L., Chautard E., Benoit C., Auboeuf D., Boland A., Battail C., Artiguenave F., Deleuze J.F. (2015). In Vitro and In Vivo Modulation of Alternative Splicing by the Biguanide Metformin. Mol. Ther. Nucleic Acids.

[18] Xie J., Ye J., Cai Z., Luo Y., Zhu X., Deng Y., Feng Y., Liang Y., Liu R., Han Z. (2020). GPD1 Enhances the Anticancer Effects of Metformin by Synergistically Increasing Total Cellular Glycerol-3-Phosphate. Cancer Res..

[19] Yue W., Zheng X., Lin Y., Yang C.S., Xu Q., Carpizo D., Huang H., DiPaola R.S., Tan X.L. (2015). Metformin combined with aspirin significantly inhibit pancreatic cancer cell growth in vitro and in vivo by suppressing anti-apoptotic proteins Mcl-1 and Bcl-2. Oncotarget.

[20] Mattick J.S. (2018). The State of Long Non-Coding RNA Biology. Noncoding RNA.

[21] Mercer T.R., Dinger M.E., Mattick J.S. (2009). Long non-coding RNAs: Insights into functions. Nat. Rev. Genet..

[22] Xing J., Liu H., Jiang W., Wang L. (2020). LncRNA-Encoded Peptide: Functions and Predicting Methods. Front. Oncol..

[23] Statello L., Guo C.J., Chen L.L., Huarte M. (2021). Gene regulation by long non-coding RNAs and its biological functions. Nat. Rev. Mol. Cell Biol..

[24] Aillaud M., Schulte L.N. (2020). Emerging Roles of Long Noncoding RNAs in the Cytoplasmic Milieu. Noncoding RNA.

[25] Fu P.F., Zheng X., Fan X., Lin A.F. (2019). Role of cytoplasmic lncRNAs in regulating cancer signaling pathways. J. Zhejiang Univ. Sci. B.

[26] Wang Y., Wang Z., Xu J., Li J., Li S., Zhang M., Yang D. (2018). Systematic identification of non-coding pharmacogenomic landscape in cancer. Nat. Commun..

[27] Pan Q., Shai O., Lee L.J., Frey B.J., Blencowe B.J. (2008). Deep surveying of alternative splicing complexity in the human transcriptome by high-throughput sequencing. Nat. Genet..

[28] Deveson I.W., Brunck M.E., Blackburn J., Tseng E., Hon T., Clark T.A., Clark M.B., Crawford J., Dinger M.E., Nielsen L.K. (2018). Universal Alternative Splicing of Noncoding Exons. Cell Syst..

[29] Yi Q.Y., Deng G., Chen N., Bai Z.S., Yuan J.S., Wu G.H., Wang Y.W., Wu S.J. (2016). Metformin inhibits development of diabetic retinopathy through inducing alternative splicing of VEGF-A. Am. J. Transl. Res..

[30] Adriaens C., Rambow F., Bervoets G., Silla T., Mito M., Chiba T., Asahara H., Hirose T., Nakagawa S., Jensen T.H. (2019). The long noncoding RNA NEAT1_1 is seemingly dispensable for normal tissue homeostasis and cancer cell growth. RNA.

[31] Zhang M., Zhao K., Xu X., Yang Y., Yan S., Wei P., Liu H., Xu J., Xiao F., Zhou H. (2018). A peptide encoded by circular form of LINC-PINT suppresses oncogenic transcriptional elongation in glioblastoma. Nat. Commun..

[32] Zhang F., Han S., Song W. (2022). Anticancer effects of metformin in experimental animal models of different types of cancer: A systematic review and meta-analysis. Lab. Anim. Res..

[33] Qin W., Zhao X., Tai J., Qin G., Yu S. (2021). Combination of Dendrobium Mixture and Metformin Curbs the Development and Progression of Diabetic Cardiomyopathy by Targeting the lncRNA NEAT1. Clinics.

[34] Schulten H.J., Bakhashab S. (2019). Meta-Analysis of Microarray Expression Studies on Metformin in Cancer Cell Lines. Int. J. Mol. Sci.

[35] Chu P.M., Yu C.C., Tsai K.L., Hsieh P.L. (2022). Regulation of Oxidative Stress by Long Non-Coding RNAs in Vascular Complications of Diabetes. Life.

[36] Cao M., Luo H., Li D., Wang S., Xuan L., Sun L. (2022). Research advances on circulating long noncoding RNAs as biomarkers of cardiovascular diseases. Int. J. Cardiol..

[37] Ghafouri-Fard S., Fathi M., Zhai T., Taheri M., Dong P. (2021). LncRNAs: Novel Biomarkers for Pancreatic Cancer. Biomolecules.

[38] Cheng Y., Wang S., Mu X. (2021). Long non-coding RNA LINC00511 promotes proliferation, invasion, and migration of non-small cell lung cancer cells by targeting miR-625-5p/GSPT1. Transl. Cancer Res..

[39] Ding J., Cao J., Chen Z., He Z. (2020). The role of long intergenic noncoding RNA 00511 in malignant tumors: A meta-analysis, database validation and review. Bioengineered.

[40] Agbana Y.L., Abi M.E., Ni Y., Xiong G., Chen J., Yun F., Yi Z., Zhang Q., Yang Z., Kuang Y. (2020). LINC00511 as a prognostic biomarker for human cancers: A systematic review and meta-analysis. BMC Cancer.

[41] Lu G., Li Y., Ma Y., Lu J., Chen Y., Jiang Q., Qin Q., Zhao L., Huang Q., Luo Z. (2018). Long noncoding RNA LINC00511 contributes to breast cancer tumourigenesis and stemness by inducing the miR-185-3p/E2F1/Nanog axis. J. Exp. Clin. Cancer Res..

[42] Luscher-Dias T., Conceicao I.M., Schuch V., Maracaja-Coutinho V., Amaral P.P., Nakaya H.I. (2021). Long non-coding RNAs associated with infection and vaccine-induced immunity. Essays Biochem..

[43] Amaral P.P., Leonardi T., Han N., Vire E., Gascoigne D.K., Arias-Carrasco R., Buscher M., Pandolfini L., Zhang A., Pluchino S. (2018). Genomic positional conservation identifies topological anchor point RNAs linked to developmental loci. Genome Biol..

[44] Winick-Ng W., Kukalev A., Harabula I., Zea-Redondo L., Szabo D., Meijer M., Serebreni L., Zhang Y., Bianco S., Chiariello A.M. (2021). Cell-type specialization is encoded by specific chromatin topologies. Nature.

[45] Gartel A.L. (2017). FOXM1 in Cancer: Interactions and Vulnerabilities. Cancer Res..

[46] Liao G.B., Li X.Z., Zeng S., Liu C., Yang S.M., Yang L., Hu C.J., Bai J.Y. (2018). Regulation of the master regulator FOXM1 in cancer. Cell Commun. Signal..

[47] Gu X., Han Y.Y., Yang C.Y., Ji H.M., Lan Y.J., Bi Y.Q., Zheng C., Qu J., Cheng M.H., Gao J. (2021). Activated AMPK by metformin protects against fibroblast proliferation during pulmonary fibrosis by suppressing FOXM1. Pharmacol. Res..

[48] Zhang B., Liu L.L., Mao X., Zhang D.H. (2014). Effects of metformin on FOXM1 expression and on the biological behavior of acute leukemia cell lines. Mol. Med. Rep..

[49] Sugiyama H., Takahashi K., Yamamoto T., Iwasaki M., Narita M., Nakamura M., Rand T.A., Nakagawa M., Watanabe A., Yamanaka S. (2017). Nat1 promotes translation of specific proteins that induce differentiation of mouse embryonic stem cells. Proc. Natl. Acad. Sci. USA.

[50] Yamanaka S., Zhang X.Y., Maeda M., Miura K., Wang S., Farese R.V., Iwao H., Innerarity T.L. (2000). Essential role of NAT1/p97/DAP5 in embryonic differentiation and the retinoic acid pathway. EMBO J..

[51] Smirnova V.V., Shestakova E.D., Nogina D.S., Mishchenko P.A., Prikazchikova T.A., Zatsepin T.S., Kulakovskiy I.V., Shatsky I.N., Terenin I.M. (2022). Ribosomal leaky scanning through a translated uORF requires eIF4G2. Nucleic Acids Res..

[52] Lee S.H., McCormick F. (2006). p97/DAP5 is a ribosome-associated factor that facilitates protein synthesis and cell proliferation by modulating the synthesis of cell cycle proteins. EMBO J..

[53] Wang Z., Ding X., Cao F., Zhang X., Wu J. (2021). Bone Mesenchymal Stem Cells Promote Extracellular Matrix Remodeling of Degenerated Nucleus Pulposus Cells via the miR-101-3p/EIF4G2 Axis. Front. Bioeng. Biotechnol..

[54] Das S., Das B. (2016). eIF4G-an integrator of mRNA metabolism?. FEMS Yeast Res..

[55] Peng W.X., Koirala P., Mo Y.Y. (2017). LncRNA-mediated regulation of cell signaling in cancer. Oncogene.

[56] Meng X., Song J., Lei Y., Zhang X., Chen Z., Lu Z., Zhang L., Wang Z. (2021). A metformin-based nanoreactor alleviates hypoxia and reduces ATP for cancer synergistic therapy. Biomater. Sci..

[57] Chen X., Li X., Zhang W., He J., Xu B., Lei B., Wang Z., Cates C., Rousselle T., Li J. (2018). Activation of AMPK inhibits inflammatory response during hypoxia and reoxygenation through modulating JNK-mediated NF-kappaB pathway. Metabolism.

[58] Gabriel M., Delforge Y., Deward A., Habraken Y., Hennuy B., Piette J., Klinck R., Chabot B., Colige A., Lambert C. (2015). Role of the splicing factor SRSF4 in cisplatin-induced modifications of pre-mRNA splicing and apoptosis. BMC Cancer.

[59] Sciarrillo R., Wojtuszkiewicz A., Assaraf Y.G., Jansen G., Kaspers G.J.L., Giovannetti E., Cloos J. (2020). The role of alternative splicing in cancer: From oncogenesis to drug resistance. Drug Resist. Updat..

[60] Toutenhoofd S.L., Foletti D., Wicki R., Rhyner J.A., Garcia F., Tolon R., Strehler E.E. (1998). Characterization of the human CALM2 calmodulin gene and comparison of the transcriptional activity of CALM1, CALM2 and CALM3. Cell Calcium.

[61] Mu G., Zhu Y., Dong Z., Shi L., Deng Y., Li H. (2021). Calmodulin 2 Facilitates Angiogenesis and Metastasis of Gastric Cancer via STAT3/HIF-1A/VEGF-A Mediated Macrophage Polarization. Front. Oncol..

[62] Claesson-Welsh L., Welsh M. (2013). VEGFA and tumour angiogenesis. J. Intern. Med..

[63] McFee R.M., Rozell T.G., Cupp A.S. (2012). The balance of proangiogenic and antiangiogenic VEGFA isoforms regulate follicle development. Cell Tissue Res..

[64] Rey C., Faustin B., Mahouche I., Ruggieri R., Brulard C., Ichas F., Soubeyran I., Lartigue L., De Giorgi F. (2016). The MAP3K ZAK, a novel modulator of ERK-dependent migration, is upregulated in colorectal cancer. Oncogene.

[65] Yong W., Yu D., Jun Z., Yachen D., Weiwei W., Midie X., Xingzhu J., Xiaohua W. (2018). Long noncoding RNA NEAT1, regulated by LIN28B, promotes cell proliferation and migration through sponging miR-506 in high-grade serous ovarian cancer. Cell Death Dis..

[66] Todorovski V., Fox A.H., Choi Y.S. (2020). Matrix stiffness-sensitive long noncoding RNA NEAT1 seeded paraspeckles in cancer cells. Mol. Biol. Cell.

[67] Liu Y., Lu F.A., Wang L., Wang Y.F., Wu C.F. (2021). Long noncoding RNA NEAT1 promotes pulmonary fibrosis by regulating the microRNA4553p/SMAD3 axis. Mol. Med. Rep..

[68] Ge Z., Yin C., Li Y., Tian D., Xiang Y., Li Q., Tang Y., Zhang Y. (2022). Long noncoding RNA NEAT1 promotes cardiac fibrosis in heart failure through increased recruitment of EZH2 to the Smad7 promoter region. J. Transl. Med..

[69] He L., Wondisford F.E. (2015). Metformin action: Concentrations matter. Cell Metab..

[70] Liu Y., Jing R., Wen Z., Li M. (2019). Narrowing the Gap Between In Vitro and In Vivo Genetic Profiles by Deconvoluting Toxicogenomic Data In Silico. Front. Pharmacol..

[71] Chau K.K., Zhang P., Urresti J., Amar M., Pramod A.B., Chen J., Thomas A., Corominas R., Lin G.N., Iakoucheva L.M. (2021). Full-length isoform transcriptome of the developing human brain provides further insights into autism. Cell Rep..

[72] Bjorklund S.S., Aure M.R., Hakkinen J., Vallon-Christersson J., Kumar S., Evensen K.B., Fleischer T., Tost J., Osbreac, Sahlberg K.K. (2022). Subtype and cell type specific expression of lncRNAs provide insight into breast cancer. Commun. Biol..

[73] Sriram A., Bohlen J., Teleman A.A. (2018). Translation acrobatics: How cancer cells exploit alternate modes of translational initiation. EMBO Rep..

[74] Zhang Z.M., Liu Z.H., Nie Q., Zhang X.M., Yang L.Q., Wang C., Yang L.L., Song G.Y. (2022). Metformin improves high-fat diet-induced insulin resistance in mice by downregulating the expression of long noncoding RNA NONMMUT031874.2. Exp. Ther. Med..

[75] Wang Y., Tang H., Ji X., Zhang Y., Xu W., Yang X., Deng R., Liu Y., Li F., Wang X. (2018). Expression profile analysis of long non-coding RNAs involved in the metformin-inhibited gluconeogenesis of primary mouse hepatocytes. Int. J. Mol. Med..

[76] Shu L., Hou X., Song G., Wang C., Ma H. (2021). Comparative analysis of long noncoding RNA expression profiles induced by resveratrol and metformin treatment for hepatic insulin resistance. Int. J. Mol. Med..

[77] Sardu C., Trotta M.C., Pieretti G., Gatta G., Ferraro G., Nicoletti G.F., Onofrio N.D., Balestrieri M.L., Amico M.D., Abbatecola A. (2021). MicroRNAs modulation and clinical outcomes at 1 year of follow-up in obese patients with pre-diabetes treated with metformin vs. placebo. Acta Diabetol..

[78] Formichi C., Fignani D., Nigi L., Grieco G.E., Brusco N., Licata G., Sabato C., Ferretti E., Sebastiani G., Dotta F. (2021). Circulating microRNAs Signature for Predicting Response to GLP1-RA Therapy in Type 2 Diabetic Patients: A Pilot Study. Int. J. Mol. Sci..

[79] Herrero-Aguayo V., Jimenez-Vacas J.M., Saez-Martinez P., Gomez-Gomez E., Lopez-Canovas J.L., Garrido-Sanchez L., Herrera-Martinez A.D., Garcia-Bermejo L., Macias-Gonzalez M., Lopez-Miranda J. (2021). Influence of Obesity in the miRNome: miR-4454, a Key Regulator of Insulin Response Via Splicing Modulation in Prostate. J. Clin. Endocrinol. Metab..

[80] Pinkney H.R., Wright B.M., Diermeier S.D. (2020). The lncRNA Toolkit: Databases and In Silico Tools for lncRNA Analysis. Noncoding RNA.

[81] Patro R., Duggal G., Love M.I., Irizarry R.A., Kingsford C. (2017). Salmon provides fast and bias-aware quantification of transcript expression. Nat. Methods.

[82] Frankish A., Diekhans M., Jungreis I., Lagarde J., Loveland J.E., Mudge J.M., Sisu C., Wright J.C., Armstrong J., Barnes I. (2021). Gencode 2021. Nucleic Acids Res..

[83] Love M.I., Soneson C., Hickey P.F., Johnson L.K., Pierce N.T., Shepherd L., Morgan M., Patro R. (2020). Tximeta: Reference sequence checksums for provenance identification in RNA-seq. PLoS Comput. Biol..

[84] Zhu A., Srivastava A., Ibrahim J.G., Patro R., Love M.I. (2019). Nonparametric expression analysis using inferential replicate counts. Nucleic Acids Res..

[85] Liberzon A., Birger C., Thorvaldsdottir H., Ghandi M., Mesirov J.P., Tamayo P. (2015). The Molecular Signatures Database (MSigDB) hallmark gene set collection. Cell Syst..

