# Supplementary material for: Metformin Treatment Modulates Long Non-Coding RNA Isoforms Expression in Human Cells
Source: Noncoding RNA. 2022 Oct 12;8(5):68. doi: 10.3390/ncrna8050068 (PMC9607547; doi:10.3390/ncrna8050068)
Supplement: Supplementary file 1 [file ncrna-08-00068-s001.zip › Supplementary_material_v2.pdf]

Supplementary material

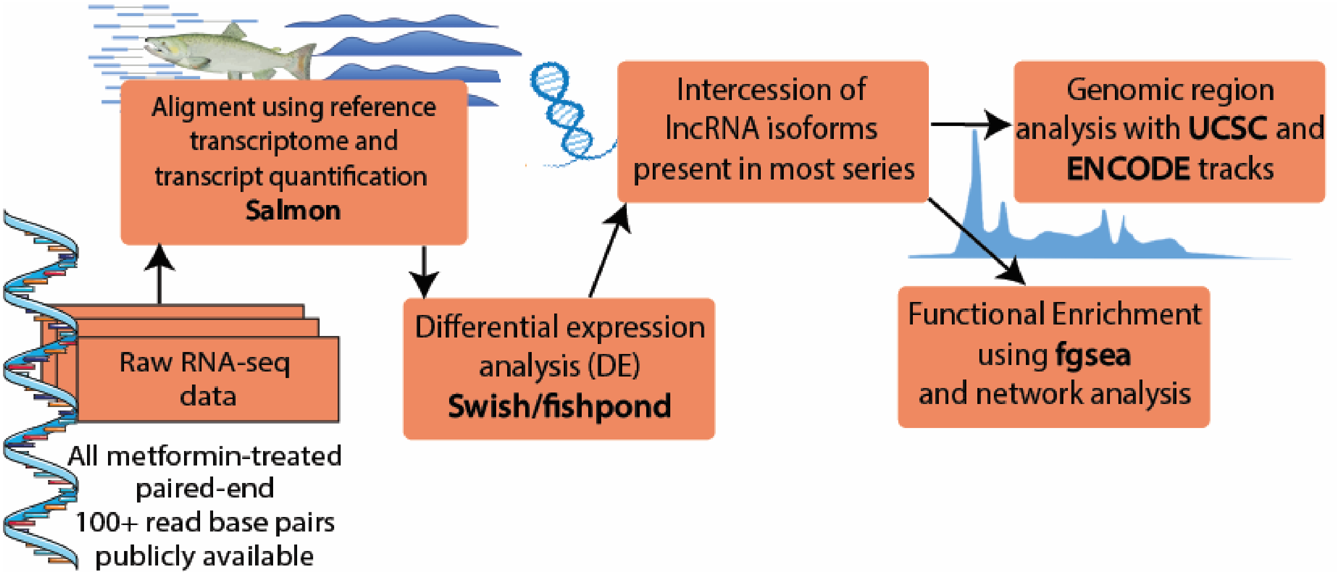

Supplementary figure S1: Global vision of the used pipeline, from the library selection to the functional annotation of lncRNA isoforms. Starting from library selection, going through pseudo-alignment in the reference transcriptome, differential expression and the intercession among series, until the functional annotation of the lncRNA isoforms

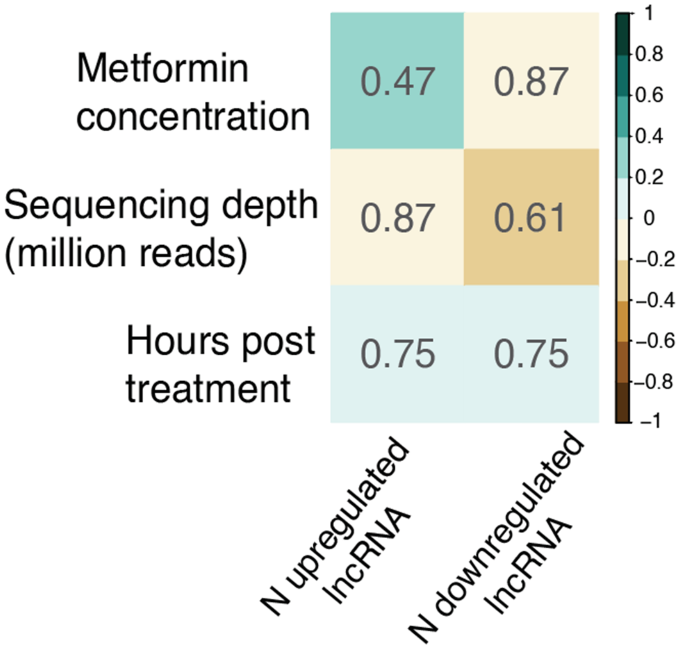

Supplementary figure S2: Pearson correlation map between number of upregulated and downregulated isoforms and other experiment characteristics. The color represents the correlation value and the number in the boxes the pvalue.

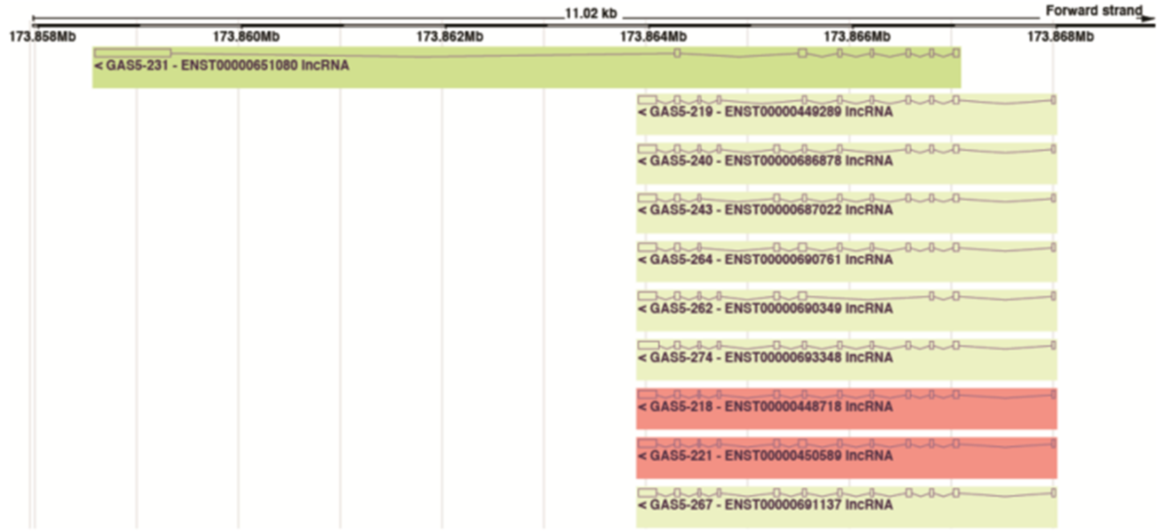

Supplementary Figure S3: *GAS5* genomic region with its transcripts from Ensembl genome Browser. In red GAS5-218 and GAS5-221 the isoforms encountered in our data. In dark green the canonical *GAS5* isoform. In green all other *GAS5* isoforms.

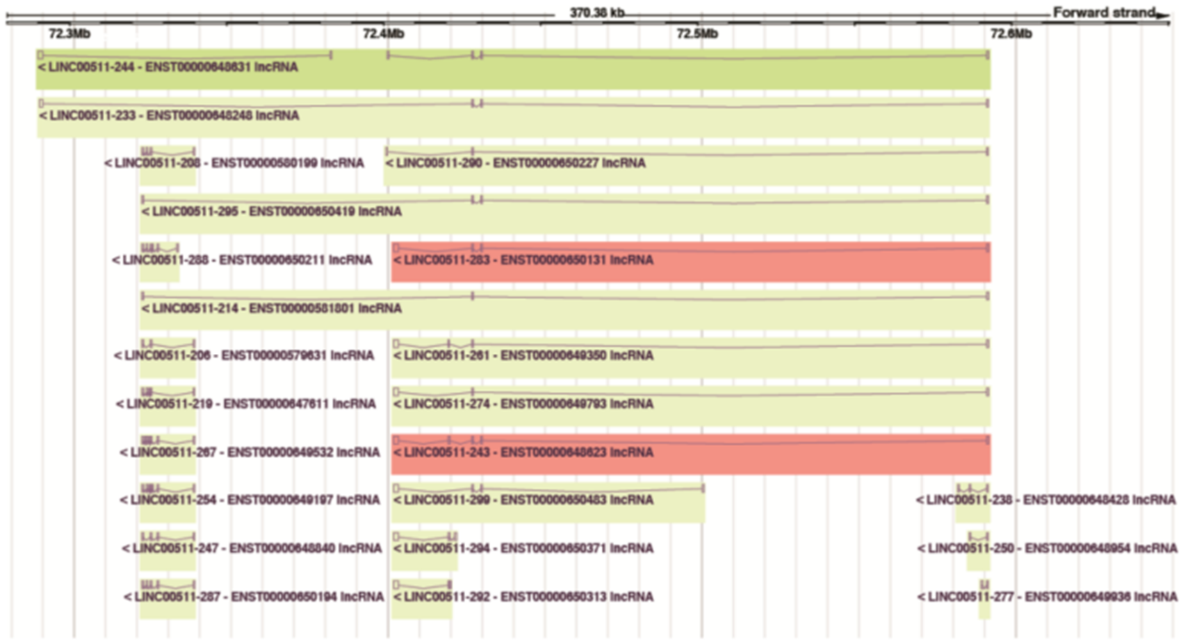

Supplementary Figure S4: *LINC00511* genomic region with some of its transcripts from Ensembl Genome Browser. In red the transcripts found in our analysis, LINC00511-243 and LINC00511-283, in dark green the canonical *LINC00511* isoform and in green the other isoforms.

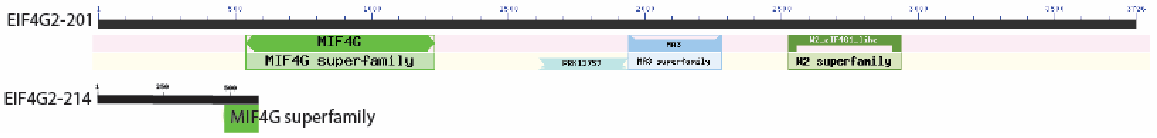

Supplementary Figure S5: Predicted domains of EIF4G2-201, the canonical EIF4G2 transcript, and EIF4G2-214, the transcript found in our analysis. Domains predicted by pfam, ncbi and CDD.
